# Real time QRS complex detection using DFA and regular grammar

**DOI:** 10.1186/s12938-017-0322-2

**Published:** 2017-02-28

**Authors:** Salah Hamdi, Asma Ben Abdallah, Mohamed Hedi Bedoui

**Affiliations:** Laboratory of Technology and Medical Imaging (LTIM), Faculty of Medicine of Monastir (FMM), University of Monastir, Monastir, Tunisia

**Keywords:** ECG signal, QRS complex, RR distance, DFA, Regular grammar

## Abstract

**Background:**

The sequence of Q, R, and S peaks (QRS) complex detection is a crucial procedure in electrocardiogram (ECG) processing and analysis. We propose a novel approach for QRS complex detection based on the deterministic finite automata with the addition of some constraints. This paper confirms that regular grammar is useful for extracting QRS complexes and interpreting normalized ECG signals. A QRS is assimilated to a pair of adjacent peaks which meet certain criteria of standard deviation and duration.

**Results:**

The proposed method was applied on several kinds of ECG signals issued from the standard MIT-BIH arrhythmia database. A total of 48 signals were used. For an input signal, several parameters were determined, such as QRS durations, RR distances, and the peaks’ amplitudes. σRR and σQRS parameters were added to quantify the regularity of RR distances and QRS durations, respectively. The sensitivity rate of the suggested method was 99.74% and the specificity rate was 99.86%. Moreover, the sensitivity and the specificity rates variations according to the Signal-to-Noise Ratio were performed.

**Conclusions:**

Regular grammar with the addition of some constraints and deterministic automata proved functional for ECG signals diagnosis. Compared to statistical methods, the use of grammar provides satisfactory and competitive results and indices that are comparable to or even better than those cited in the literature.

## Background

The sequence of Q, R, and S peaks complex detection is one of the most frequently addressed tasks in ECG signal processing and analysis. A wide range of methods allowing high detection rates have been proposed and used [1–4]. Nevertheless, the problem remains open given the variety of ECG signals and the noise that might impact them. These methods include the Support Vector Machine (SVM) [5–12], the fuzzy neural networks [13–16] and the wavelets [1, 17–24]. Sahambi et al. [25, 26] utilized the first order derivative of the Gaussian function as a wavelet for the characterization of ECG beats. The author used the dyadic wavelet transform to detect and measure the different parts of a signal, especially the location of the beginning and the end of the QRS complex. Sahambi et al. showed the robustness of the algorithm in the presence of a high frequency noise added to the signal. In [1], a dyadic wavelet transform was used to extract the characteristics of the ECG signal. The algorithm detected the QRS complex and the T wave, and then the P wave. Gramatikov et al. [27] focused on the morphology of the QRS complex and used the Morlet wavelet transform for the analysis of ECG recordings in patients with left or right coronary stenosis. The detection of the QRS complexes can be performed by a simple thresholding of the signal in terms of amplitude as the R peaks are generally larger than the other waves. The amplitude of the T wave is sometimes similar to that of the R peak, which can cause errors in the final result and the detection rate.

Several QRS-complex-research algorithms based extensively on the proportionately high amount of QRS energy [28] were used. Most algorithms were based on the application of neural networks, hidden Markov model, syntactic methods, etc. [29–40]. More details on the QRS complex detection techniques, comparing their effectiveness and their calculation complexities, can be found in the presence of artifacts. Generally, the QRS detection algorithms are based on one of the temporal derivatives of methods, wavelets, filter banks and mathematical morphology [41–45]. These approaches are very effective and have a high accuracy rate that exceeds 99%. Kohler et al. [46] established a detailed study summarizing the different techniques for QRS detection. The discussed methods were sorted by categories and their performance was compared. Dotsinsky et al. [47] developed a heuristic algorithm applied on two channel recordings from AHA and MIT-BIH Arrhythmia Database.

Few approaches were based on the grammatical formalism [48]. Gao et al. [49] affirmed that the use of grammar, compared to statistical methods, provides more flexibility in applications. The syntactic approaches can efficiently represent the signal structures and consequently facilitate data retrieval by means of their structures. The main advantage of these methods is that the representation is concise. The syntactic approaches can better represent the ECG structures and therefore facilitate information recovery. As grammar clearly represents hierarchical structures using non-terminal and terminal nodes, the input data seem to be a structured scene having a hierarchical order. Moreover, the syntactic approaches can describe a large set of complex patterns utilizing small sets of simple primitives and grammatical rules. Kokai et al. [50] used grammar for QRS complex classification and distinction between QRS and non-QRS patterns. Panagiotis et al. [51] applied a syntactic method for ECG recognition and the measurement of the associated parameters. However, those methods were very sensitive to noise. Several morphologies generated erroneous peaks and thus hindered the grammatical description of the signal. The authors also did not use the grammar formalism during the extraction phase of the peaks. Peak recognition was performed using another method independently of grammar. Hamdi et al. [52] presented a context-free grammar to describe an entire ECG signal. However, context-free grammar could not represent all the different kinds of ECG signals. The author focused only on normal cases and the method was applied on signal of short durations. Furthermore, the author compared his method with the old techniques of Holsinger [53] and Fraden and Neuman [54]. Hanieh et al. [55] proposed a method to detect atrial arrhythmia. The suggested method modelled arrhythmia by a regular expression. The input signal was transformed into a character string in which each character represented an ECG signal component. Different experiments on MIT-BIH arrhythmia database show the efficiency of the method and the detection algorithm compared to conventional approaches. However, this algorithm has a sensitivity rate that does not exceed 96.3%.

The present work is based on learning automata to recognize rest phases, negative and positive peaks. The QRS complex was described by automation devices. Several parameters were determined, such as the number of QRS complexes, the QRS durations, the RR distances and the amplitudes of peaks.

The remainder of the paper is organized as follows. “Methods” section explains the material and the proposed method. “Results and discussion” section presents and discusses the obtained results, and a comparative study in terms of sensitivity rates was performed on several statistical methods. “Conclusion” section concludes the paper.

## Methods

### Method overview

The suggested method recognizes the QRS complex in an ECG waveform based on grammar formalism. The grammatical formalism can efficiently represent the ECG and consequently facilitate the retrieval of signal features. The main advantage of this method allows a representation of several QRS in a concise way. It can better represent the QRS complex structures and therefore facilitate the recovery of several parameters. As regular grammar clearly represents hierarchical structures using a set of symbols and regular expressions, he ECG input seems to be a structured scene having a hierarchical order. Furthermore, the proposed method can describe a large set of QRS complexes using sets of simple primitives, grammatical rules, and deterministic finite automata.

**Fig. 1:**
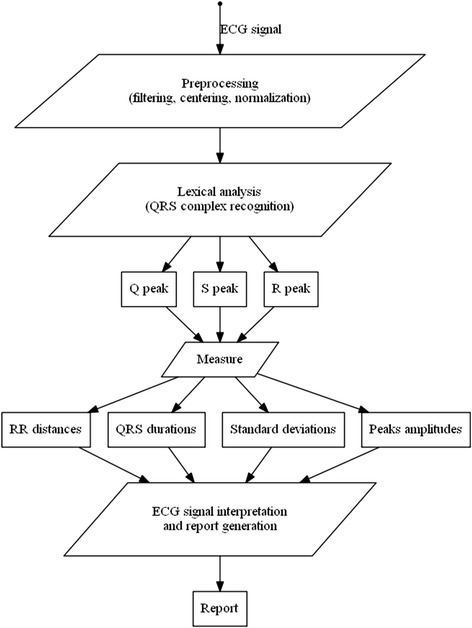
An overview of the proposed method

Figure 1 summarizes all the steps. The input signal amplitude is filtered, centralized and normalized. Then, the lexical analysis step recognizes tokens including positive and negative peaks. A QRS complex is assimilated to a pair of adjacent peaks that satisfy certain criteria of standard deviation. It is described using deterministic automata and regular expressions. Finally, the analyzer computes the RR distances, the complex-QRS durations, the standard deviation of RR distances, the standard deviation of QRS durations, and generates a report according to sampling frequency, time and amplitude values.

An ECG signal S[n] is actually too noisy and contains many artifacts, hence the need for preprocessing phases to reduce noise and facilitate lexical analysis afterwards. The band-pass filter reduces the influence of muscle noise, 60 Hz interference, base line wander, and T wave interference. The desirable pass-band to maximize the QRS energy is approximately 5–15 Hz [30].

**Fig. 2:**
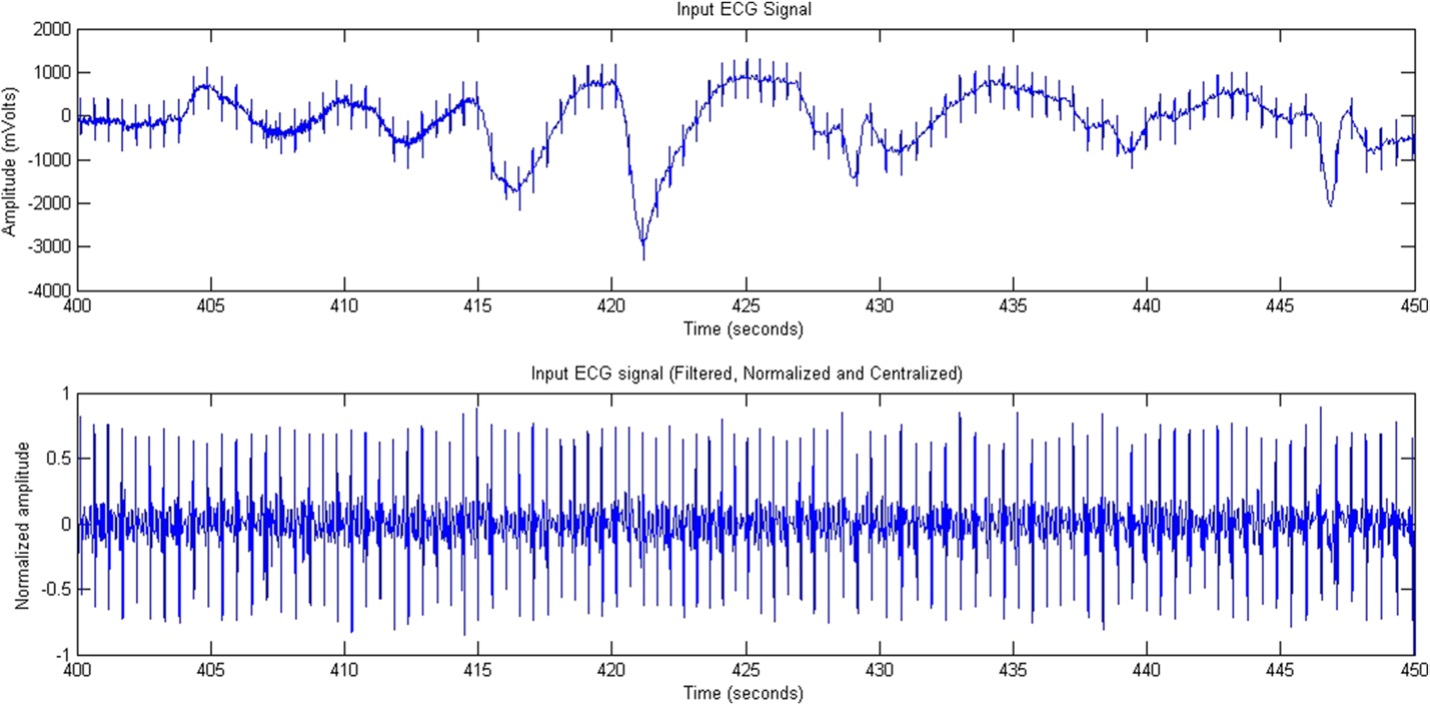
A normalized and centered ECG signal representing tachycardia. The signal was filtered by a band-Pass filter

The following mathematical equations describe the various steps of the preprocessing phase: band-pass filtering, signal centering, and normalization of signal amplitude. An example is displayed later in Fig. 2 where a normalized and centered ECG signal representing a tachycardia is filtered by a band-Pass filter.

Step 1: Band-pass filtering of the signal S[n] where H[n] is a band-pass filter and 5–15 Hz is the cutoff frequency.

1 $${\text{S}}1\left[ {\text{n}} \right] = {\text{S}}\left[ {\text{n}} \right] * {\text{H}}[{\text{n}}]$$

Step 2: Signal centering:

2 $${\text{S}}2\left[ {\text{n}} \right] = {\text{S}}1\left[ {\text{n}} \right] - \frac{{\mathop \sum \nolimits_{{{\text{i}} = 1}}^{\text{m}} {\text{S}}1[{\text{i}}]}}{\text{m}}$$

The m parameter is the signal length.

Step 3: Amplitude signal normalization:

3 $${\text{S}}3\left[ {\text{n}} \right] = \frac{{{\text{S}}2\left[ {\text{n}} \right] - {\text{Mean }}({\text{S}}2\left[ {\text{n}} \right])}}{{{\text{Max}}\,({\text{S}}2\left[ {\text{n}} \right] - {\text{Mean }}({\text{S}}2[{\text{n}}]))}}$$

Figure 2 presents an example of a real ECG signal before and after the filtering process. The input signal was issued from one patient with tachycardia. Preprocessing did eliminate the artifacts and centralize the signal.

### Grammatical analysis of the signal

The output signal amplitude is processed in the form of a value sequence belonging to the bounded interval [−1, 1]. The normalized amplitude is described as a sequence of almost nil, negative and positive values; i.e., the signal is assimilated to a language where the QRS complex represents a suite of lexemes.

The alphabet ∑ = {0,1,2,3,4,5,6,7,8,9, -,.} contains all symbols that can represent a normalized amplitude belonging to the bounded interval [−1, 1]. Then, the regular expressions make the lexical analysis of the signal. In fact, the deterministic automata and the regular expressions represent the rest phase, the positive peak and the negative peak, and make up the QRS complex with the addition of some constraints of standard deviation.

Mathematically, a positive or negative peak must show a higher standard deviation σ that is much greater than a threshold σ1.

Given the sampling frequency *Fe*, a peak, a wave or a rest phase are made of a sequence of *k* normalized simples {*a*_*1*_ *, a*_*2*_ *,…, a*_*k*_} having an average amplitude ${\bar{\text{a}}}$ The calculation of the standard deviation σ and the duration Δ are as follows:

4 $$\upsigma = \sqrt {\sum\limits_{{{\text{i}} = 1}}^{\text{k}} {\frac{{\left( {{\text{a}}_{\text{i}} - {\bar{\text{a}}}} \right)^{2} }}{\text{k}}} }$$

5 $${\bar{\text{a}}} = \mathop \sum \limits_{{{\text{i}} = 1}}^{\text{k}} \frac{{{\text{a}}_{\text{i}} }}{\text{k}}$$

6 $$\Delta = \frac{\text{k}}{\text{Fe}}$$

**Fig. 3:**
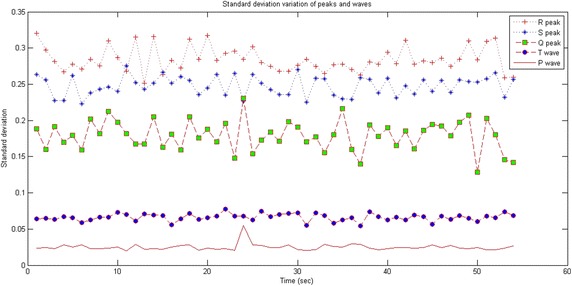
Standard deviation variation of *peaks* and *waves*

Figure 3 plots the standard deviations of several Q, R and S peaks as well as P and T waves. Figure 3 confirms that both R and S peaks show very important standard deviations that are higher than 0.2. The Q peak has standard deviations that are higher than 0.1 while both P and T waves have very low values of standard deviations below 0.05.

According to Fig. 3, σ1 = 0.1. Starting from this value, we can distinguish between the peaks and the waves. Actually, a QRS complex is assimilated to a pair of adjacent peaks that satisfy the criteria of standard deviation.

**Fig. 4:**
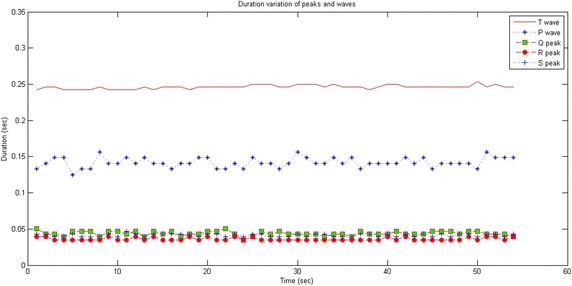
Duration variation of *peaks* and *waves*

Figure 4 plots the durations of several Q, R and S peaks as well as P and T waves for several ECG signal recordings. This confirms that the durations of these peaks are small and shorter than 0.1 s while both P and T waves’ durations are longer than 0.1 s.

Based on Fig. 4, Δ1 = 0.1 s is defined as a threshold. Starting from this value, we can distinguish between the peaks and the waves. Thus, a QRS complex is assimilated to a pair of adjacent peaks that satisfy the criteria of standard deviation and duration.

A Deterministic Finite Automaton (DFA) on an alphabet Σ is a quadruple (Q, δ, q0, F) where:

- q0 is the start state.
- Q is a finite set of states.
- F is a part of Q called final states.
- δ is a transition function Q × Σ in Q.

The DFA consists of a finite set of states (often denoted Q), a finite set Σ of symbols (alphabet), a transition function that takes as argument a state and a symbol and returns a state (often denoted δ), a start state often denoted q0, and a set of final or accepting states (often denoted F). We have q0 ∈ Q and F ⊆ Q.

Grammatically, the symbol ‘∊’ means an empty word having zero length, ‘*’ means ‘zero or more times’, ‘+’ means ‘one or more times’, and the symbol ‘?’ means ‘zero or one time’.

**Fig. 5:**
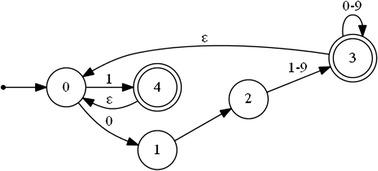
A deterministic automaton representing a normalized positive peak (R)

The following regular expression and the deterministic automaton (Fig. 5) describe a normalized positive peak:

7 $${\text{R }} = \, \left\{ {0.\left[ { 1{-} 9} \right]\left[ {0 - 9} \right]*| 1} \right\} +$$

8 $$\sigma {\text{R }} > \, \sigma 1$$

9 $$\Delta {\text{R }} < \, \Delta 1$$

The start sate q0 = {0}.

The finite set of states Q = {0,1,2,3,4}.

The final set of states F = {3,4}.

The transition functions are:

**Table:** 

δ(0,0) = 1	δ(1,·) = 2	δ(2,1–9) = 3	δ(3,0–9) = 3
δ(3,ε) = 0	δ(0,1) = 4	δ (4, ε) = 0	

**Fig. 6:**
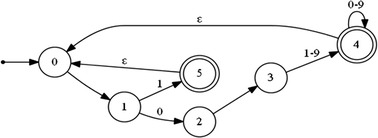
A deterministic automaton representing a normalized negative peak (Q or S)

The following regular expression and the deterministic automaton (Fig. 6) describe a normalized negative peak:

10 $${\text{Q }} = \, \left\{ { - \, 0.\left[ { 1{-} 9} \right]\left[ {0 - 9} \right]*| - 1} \right\} +$$

11 $$\sigma {\text{Q }} > \frac{\sigma 1}{2}$$

12 $$\Delta {\text{Q}} < \, \Delta 1$$

13 $${\text{S }} = \, \left\{ { \, - 0.\left[ { 1{-} 9} \right]\left[ {0 - 9} \right]*| - 1} \right\} +$$

14 $$\sigma {\text{S }} > \, \sigma 1$$

15 $$\Delta {\text{S}} < \, \Delta 1$$

The start sate q0 = {0}.

The finite set of states Q = {0,1,2,3,4,5}.

The final set of states F = {4,5}.

The transition functions are:

**Table:** 

δ(0,−) = 1	δ(1,0) = 2	δ(2,·) = 3	δ(3,1–9) = 4
δ(4,0–9) = 4	δ(4,ε) = 0	δ(1,1) = 5	δ (5, ε) = 0

**Fig. 7:**
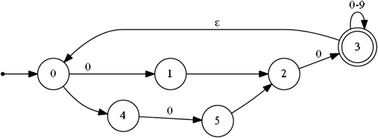
A deterministic automaton representing a normalized and short rest phase separating the peaks

The following regular expression and the deterministic automaton (Fig. 7) describe a normalized and short rest phase separating the peaks:

The start sate q0 = {0}.

The finite set of states Q = {0, 1, 2, 3, 4, 5}.

The final state F = {3}.

The transition functions are:

**Table:** 

δ(0,0) = 1	δ(1,·) = 2	δ(2,0) = 3	δ(3,0–9) = 3
δ(3,ε) = 0	δ(0,−) = 4	δ(4,0) = 5	δ(5,·) = 2

16 $${\text{rest}} = \, \left\{ {\left\{ - \right\}? \, 0.0\left[ {0 - 9} \right]*} \right\} +$$

17 $$\Delta {\text{rest}} < \frac{\Delta 1}{2}$$

**Fig. 8:**
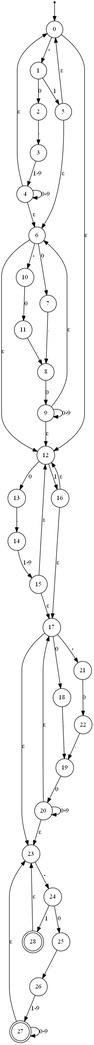
A deterministic automaton representing an entire normalized QRS complex

The regular expression below and Fig. 8 describe a normalized QRS complex. Q is the first peak pointing down, which is not always visible on the plot. The R peak is the second one. It is of high amplitude and directed upward. The S peak is the last one, and it is directed downward.

18 $${\text{QRS}} = \left\{ {\text{Q}} \right\}?\left\{ {\text{rest}} \right\}?\left\{ {\text{R}} \right\}\left\{ {\text{rest}} \right\}?\left\{ {\text{S}} \right\}$$

The start sate q0 = {0}.

The finite set of states Q = {0,1,2,3,..,27,28}.

The final set of states F = {27,28}.

The transition functions are:

**Table:** 

δ(0,·) = 1	δ(1,0) = 2	δ(2,·) = 3	δ(3,1–9) = 4
δ(4,0–9) = 4	δ(4,ε) = 6	δ(1,1) = 5	δ(4,ε) = 0
δ(5,ε) = 6	δ(5,ε) = 0	δ(6,0) = 7	δ(7,·) = 8
δ(8,0) = 9	δ(6,·) = 10	δ(10,0) = 11	δ(11,·) = 8
δ(9,0–9) = 9	δ(9,ε) = 6	δ(6,ε) = 12	δ(0,ε) = 12
δ(9,ε) = 12	δ(12,0) = 13	δ(13,·) = 14	δ(14,1–9) = 15
δ(15,ε) = 17	δ(15,ε) = 12	δ(12,1) = 16	δ(16,ε) = 12
δ(16,ε) = 17	δ(17,0) = 18	δ(18,.) = 19	δ(19,0) = 20
δ(20,0–9) = 20	δ(17,·) = 21	δ(21,0) = 22	δ(22,·) = 19
δ(20,ε) = 17	δ(17,ε) = 23	δ(20,ε) = 23	δ(23,·) = 24
δ(24,0) = 25	δ(25,·) = 26	δ(26,1–9) = 27	δ(27,ε) = 23
δ(27,0–9) = 27	δ(24,1) = 28	δ(28,ε) = 23	

Grammatically, QRS is assimilated to a suite of negative and positive peaks which may be separated by a very short resting phase. It should be noted that the above regular expression and the deterministic automaton presume that the Q peaks and the rest phases may be absent.

## Results and discussion

### Results

In this section, the method described above was applied on several real ECG signals representing different patients and issued from the standard MIT-BIH arrhythmia database. For all the input signals, the QRS complexes were detected, the Q, R and S peaks were separated and the RR distances were measured. The RR distance refers to the duration between two successive R peaks. Furthermore, a comparative study with several methods [30, 37, 38, 44, 56–64] was performed with regard to QRS complex detection.

Table 1 shows an application on several real ECG signals to extract the QRS complex. The True Positive (TP), the False Positive (FP), the False Negative (FN), the Sensitivity (Se), the specificity (Sp), the False Detection Rate (FDR), and the False Negative Rate (FNR) values are determined where:

**Table 1:** Application on several real ECG signals issued from the standard database MIT-BIH, extraction of QRS complex, sensitivity and specificity rates determination

Record	Record length	Real number of QRS	TP	FN	FP	Se (%)	Sp (%)	FDR (%)	FNR (%)	$\overline{\text{RR}}$	σRR	$\overline{\text{QRS}}$	σQRS
100	1805	2273	2272	1	0	99.96	100.00	0.00	0.04	0.79	0.05	0.05	0.00
101	1805	1865	1864	1	0	99.95	100.00	0.00	0.05	0.96	0.07	0.06	0.00
102	1805	2187	2183	4	2	99.82	99.91	0.09	0.18	0.83	0.09	0.14	0.01
103	1805	2084	2082	2	2	99.90	99.90	0.10	0.10	0.86	0.05	0.05	0.00
104	1805	2230	2211	19	24	99.15	98.93	1.07	0.85	0.81	0.08	0.04	0.04
105	1805	2572	2571	1	0	99.96	100.00	0.00	0.04	0.70	0.11	0.07	0.00
106	60	67	67	0	0	100.00	100.00	0.00	0.00	0.88	0.09	0.06	0.00
107	60	70	70	0	2	100.00	97.22	2.78	0.00	0.82	0.12	0.12	0.02
108	1805	1763	1761	2	0	99.89	100.00	0.00	0.11	1.02	0.21	0.09	0.08
109	1805	2532	2526	6	2	99.76	99.92	0.08	0.24	0.71	0.05	0.09	0.00
111	60	69	69	0	0	100.00	100.00	0.00	0.00	0.89	0.15	0.05	0.01
112	1805	2539	2537	2	6	99.92	99.76	0.24	0.08	0.71	0.03	0.06	0.00
113	1805	1794	1794	0	1	100.00	99.94	0.06	0.00	1.00	0.09	0.05	0.00
114	60	54	54	0	0	100.00	100.00	0.00	0.00	1.10	0.04	0.03	0.00
115	1805	1953	1953	0	0	100.00	100.00	0.00	0.00	0.92	0.08	0.05	0.00
116	60	78	78	0	0	100.00	100.00	0.00	0.00	0.76	0.01	0.06	0.00
117	1805	1535	1534	1	1	99.93	99.93	0.07	0.07	1.17	0.05	0.06	0.00
118	1805	2275	2275	0	12	100.00	99.48	0.52	0.00	0.78	0.10	0.07	0.00
119	1805	1987	1987	0	0	100.00	100.00	0.00	0.00	0.90	0.25	0.07	0.02
121	1805	1863	1861	2	3	99.89	99.84	0.16	0.11	0.96	0.09	0.08	0.00
122	1805	2476	2475	1	2	99.96	99.92	0.08	0.04	0.72	0.04	0.07	0.00
123	1805	1518	1517	1	4	99.93	99.74	0.26	0.07	1.18	0.12	0.06	0.00
124	60	49	49	0	0	100.00	100.00	0.00	0.00	1.21	0.02	0.07	0.01
200	60	87	87	0	0	100.00	100.00	0.00	0.00	0.72	0.40	0.09	0.01
201	60	90	90	0	0	100.00	100.00	0.00	0.00	0.66	0.13	0.06	0.00
202	1805	2136	2111	25	0	98.83	100.00	0.00	1.17	0.85	0.30	0.07	0.00
203	60	97	97	0	0	100.00	100.00	0.00	0.00	0.61	0.24	0.08	0.01
205	60	89	89	0	0	100.00	100.00	0.00	0.00	0.66	0.01	0.05	0.00
207	1805	1862	1859	3	0	99.84	100.00	0.00	0.16	0.96	0.21	0.07	0.09
208	60	87	87	0	0	100.00	100.00	0.00	0.00	0.69	0.26	0.07	0.02
209	1805	3004	3002	2	7	99.93	99.77	0.23	0.07	0.59	0.08	0.05	0.00
210	1805	2647	2606	41	9	98.45	99.66	0.34	1.55	0.69	0.13	0.07	0.01
212	1805	2748	2748	0	5	100.00	99.82	0.18	0.00	0.65	0.04	0.06	0.00
213	1805	3251	3243	8	2	99.75	99.94	0.06	0.25	0.55	0.04	0.06	0.01
214	1805	2262	2229	33	0	98.54	100.00	0.00	1.46	0.81	0.22	0.07	0.00
215	1805	3363	3337	26	0	99.23	100.00	0.00	0.77	0.54	0.09	0.06	0.00
217	1805	2208	2206	2	0	99.91	100.00	0.00	0.09	0.88	0.25	0.10	0.01
219	1805	2154	2152	2	0	99.91	100.00	0.00	0.09	0.83	0.22	0.06	0.00
220	1805	2048	2047	1	4	99.95	99.80	0.20	0.05	0.88	0.09	0.05	0.00
221	1805	2427	2400	27	0	98.89	100.00	0.00	1.11	0.75	0.20	0.06	0.01
222	60	75	75	0	0	100.00	100.00	0.00	0.00	0.81	0.12	0.05	0.00
223	60	80	80	0	0	100.00	100.00	0.00	0.00	0.75	0.08	0.07	0.00
228	60	68	68	0	0	100.00	100.00	0.00	0.00	0.86	0.27	0.07	0.01
230	1805	2256	2219	37	0	98.36	100.00	0.00	1.64	0.81	0.18	0.06	0.00
231	60	63	63	0	0	100.00	100.00	0.00	0.00	0.94	0.11	0.06	0.00
232	1805	1780	1747	33	4	98.15	99.77	0.23	1.85	1.03	0.65	0.06	0.00
233	60	94	94	0	0	100.00	100.00	0.00	0.00	0.58	0.13	0.07	0.01
234	1805	2753	2752	1	0	99.96	100.00	0.00	0.04	0.65	0.03	0.06	0.00
Total	58,720	7,3562	73,278	284	92	99.74	99.86	0.14	0.26	0.82	0.13	0.07	0.01

The average sensitivity (Se) rate of the proposed method was 99.74% and the average specificity (Sp) rate was 99.86%. The average False Detection Rate (FDR) rate and the average False Negative Rate (FNR) rate were 0.14 and 0.26% respectively

TP represents the correctly identified QRS.

FP represents the incorrectly identified QRS.

FN represents the incorrectly rejected QRS.

19 $${\text{Sensitivity }}\left( \% \right) = \frac{\text{TP}}{{{\text{TP}} + {\text{FN}}}} *100$$

20 $${\text{Specificity}}\left( {\text{\%}} \right) = \frac{\text{TP}}{{{\text{TP}} + {\text{FP}}}} *100$$

21 $${\text{False detection rate }}\left( {\text{\%}} \right) = \frac{\text{FP}}{{{\text{TP}} + {\text{FP}}}} *100$$

22 $${\text{False negative rate }}\left( {\text{\%}} \right) = \frac{\text{FN}}{{{\text{TP}} + {\text{FN}}}} *100$$

For each recording, the standard deviation of the RR distances denoted σRR and the standard deviation of the QRS durations denoted σQRS were computed. It should be noted that the standard deviation parameters are a sign of relationship between the obtained values and the average value where the *n* parameter is the total number of RR distances:

23 $$\upsigma{\text{RR}} = \sqrt {\sum\limits_{{{\text{i}} = 1}}^{\text{n}} {\frac{{({\text{RR}}_{\text{i}} - \overline{\text{RR}}) }}{\text{n}}} }$$

24 $$\overline{\text{RR}} = \mathop \sum \limits_{{{\text{i}} = 1}}^{\text{n}} \frac{{{\text{RR}}_{\text{i}} }}{\text{n}}$$

25 $$\upsigma{\text{QRS}} = \sqrt {\sum\limits_{{{\text{i}} = 1}}^{{{\text{n}} + 1}} {\frac{{\left( {{\text{QRS}}_{\text{i}} - \overline{\text{QRS}} } \right)^{2} }}{{{\text{n}} + 1}}} }$$

26 $$\overline{\text{QRS}} = \mathop \sum \limits_{{{\text{i}} = 1}}^{{{\text{n}} + 1}} \frac{{{\text{QRS}}_{\text{i}} }}{{{\text{n}} + 1}}$$

σRR and σQRS parameters were added to quantify the regularity of RR distances and QRS durations, respectively. A short σRR meant that all the RR distances were stable. A short σQRS meant that all the QRS durations were also stable.

In order to validate the proposed method, we used several kinds of ECG signals issued from the MIT-BIH arrhythmia database. These signals had a 360 Hz sampling frequency, a 200 gain and a 1024 mV base. For each input signal, several parameters were determined, such as the number of QRS, the RR distances, the QRS durations, the standard deviation of RR distances, the standard deviation of QRS durations, and the peaks amplitudes (Table 1).

According to these results, a σRR lower than 0.1 meant that all the RR distances were regular. However, a σRR higher than 0.1 meant that the obtained values of the RR distances were irregular. Similarly, a short σQRS lower than 0.1 meant that all the QRS durations were regular and a high σ*Q*RS more than 0.1 implied that the obtained values were irregular.

**Fig. 9:**
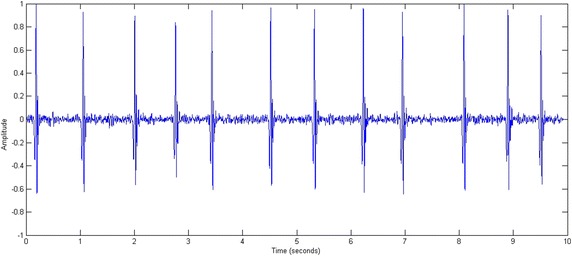
A portion of a normalized ECG representing an irregular beat rate

**Fig. 10:**
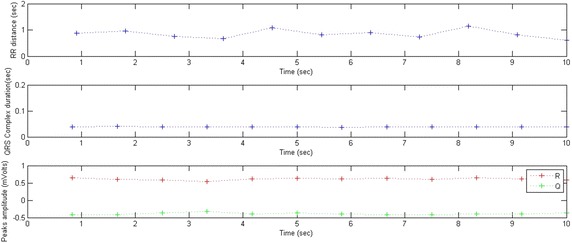
RR distances, QRS durations and peaks amplitudes variations; $\overline{RR} = \, 0. 8 4 {\text{ s}}$, σRR = 0.15, $\overline{QRS} = 0.0 3 {\text{ s}}$, σQRS = 0.01

Figures 9 and 10 show the result obtained from a portion of an ECG representing an irregular beat rate. The various indicators of the signal (RR distance; QRS complex; Q, R and S amplitudes) are displayed. The average RR and QRS values are 0.84 and 0.03 s respectively. However, the RR distances are irregular. In fact, the standard deviation of the RR distances is σRR = 0.15. This high value proves that the RR distance is not stable.

The QRS complexes have regular durations of less than 0.1 s. Indeed, the standard deviation of the QRS durations is σQRS = 0.01. In this case, this low value indicates that the QRS duration is stable.

**Fig. 11:**
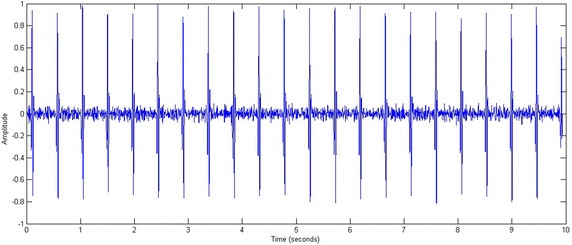
A portion of a normalized ECG representing a regular beat rate

**Fig. 12:**
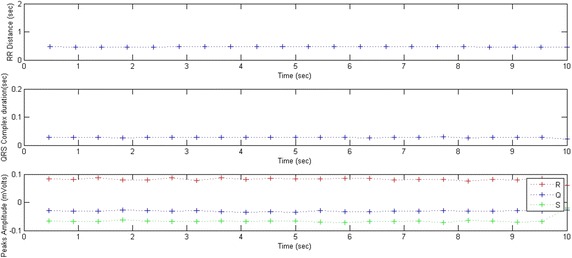
RR distances, QRS durations and peaks amplitudes variations; $\overline{RR} = \, 0. 4 6$, σRR = 0.00, $\overline{QRS} = \, 0.0 2$, σQRS = 0.00

Figures 11 and 12 show the results obtained from an ECG portion representing a regular beat rate. The average RR and QRS values are 0.46 and 0.02 s respectively. The RR distances are regular and the standard deviation of the RR distances is σRR = 0.00. This low value shows that the RR distance is stable.

The QRS complexes have regular durations of less than 0.1 s, the standard deviation of the QRS durations being σQRS = 0.00. This low value indicates that the QRS duration is stable.

### Noise sensitivity

In this section, we examined the present method’s sensitivity to noise by adding a different noise value to the ECG recordings. Table 2 shows the variation of sensitivity and specificity rates according to Signal-to-Noise Ratio (SNR).

**Table 2:** Performance of the proposed method on ECG signals with different SNR values

Record	SNR (dB)	90	80	70	60	50	40	30	25	24	23	22	21	20
100	Se (%)	99.96	99.96	99.96	99.96	99.96	99.96	99.96	93.27	90.32	85.97	79.94	63.57	55.52
	Sp (%)	100.0	100.0	100.0	100.0	100.0	100.0	99.91	83.73	80.29	76.99	72.80	62.72	57.52
101	Se (%)	99.95	99.95	99.95	99.95	99.95	99.95	99.79	95.28	86.27	84.13	81.88	87.56	48.04
	Sp (%)	100.0	100.0	100.0	100.0	100.0	100.0	99.95	84.86	69.44	66.65	65.37	69.05	39.54
102	Se (%)	99.82	99.82	99.82	99.82	99.82	97.49	91.13	83.36	79.84	74.67	66.03	57.80	43.80
	Sp (%)	99.91	99.91	99.91	99.91	99.91	96.22	88.90	83.51	75.52	68.84	58.87	51.34	40.47
103	Se (%)	99.90	99.90	99.90	99.90	99.90	99.90	99.86	97.46	95.30	88.58	83.25	70.59	54.89
	Sp (%)	99.90	99.90	99.90	99.90	99.90	99.90	99.90	95.85	92.07	81.75	74.95	60.46	45.52
105	Se (%)	99.96	99.96	99.96	99.96	99.96	98.41	97.51	93.58	90.90	81.22	73.21	73.13	53.30
	Sp (%)	100.0	100.0	100.0	100.0	100.0	99.10	99.09	95.90	92.56	79.61	73.15	73.04	61.01
Total	Se (%)	99.92	99.92	99.92	99.92	99.92	99.14	97.65	92.59	88.53	82.91	76.86	70.53	51.11
	Sp (%)	99.97	99.97	99.97	99.97	99.97	99.20	97.95	88.12	79.89	73.42	68.42	64.28	47.27

For SNR values greater than 40 dB, the method provided high sensitivity values that exceeded 99%. For SNR values greater than 30 dB, the method yielded sensitivity values which exceeded 97%. For the SNR values that were lower than 24 dB, the sensitivity value decreased to 90%.

**Fig. 13:**
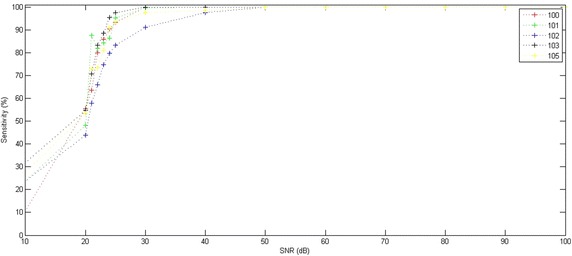
Sensitivity variation according to Signal-to-Noise Ratio (SNR). Application on 100, 101, 102, 103 and 105 ECG records

Figure 13 shows the variation sensitivity rate depending on the SNR for different ECG recordings issued from the MIT-BIH database (100, 101, 102, 103 and 105 records). For the SNR values lower than 20 dB, the method provided sensitivity rates lower than 50%.

Generally, sensitivity becomes increasingly important where SNR values are greater than 30 dB. For SNR values exceeding 30 dB, the method gave sensitivity rates which exceeded 97%. When SNR exceeded 40 dB, the method provided high sensitivity values that reached 99%.

### Comparison of performance

In order to compare the detection algorithm with other works in the literature, the quality performance detection was compared with several algorithms tested and validated on the MIT-BIH data base. Those algorithms varied and each one was based on an appropriate technique. Table 3 shows a comparative study with several methods [30, 37, 38, 44, 56–64] applied on the same MIT-BIH database in terms of sensitivity rates.

**Table 3:** Comparison of performance of several QRS detection algorithms cited in the literature

Method	Method description	Sensitivity (%)
Pan et al. [30]	Derivative approach based on filtering and analyzing the slope	99.30
Szu et al. [37]	Neural network based on adaptive filtering	99.50
Sai et al. [38]	Using the Euclidean distance metric with KNN algorithm (K-Nearest Neighbor)	99.81
Ben et al. [44]	Approach based on discrete wavelet decomposition and calculation of energy	99.39
Ham et al. [56]	Derivative approach based on filtering using an optimized process of rule decision	99.46
Cho et al. [57]	A multi wavelet packet decomposition	99.14
Had et al. [58]	Empirical modal decomposition (EMD)	99.92
Chr et al. [59]	Use of adaptive thresholding	99.65
Gha et al. [60]	Mathematical model based on the continuous wavelet transform (CWT)	99.91
Kry et al. [61]	Technique based on the recursive temporal prediction	99.00
Meh et al. [62]	Approach based on SVM (Support Vector Machine)	99.75
Gri et al. [63]	A transformation based on the duration and the energy	99.26
Tra et al. [64]	Approach based on mathematical morphology	99.38
The suggested method	Approach based on regular grammar and calculation of the standard deviation	99.74

Based on the results presented in Table 3, all the above mentioned algorithms have good QRS complex detection capability with a sensitivity that exceeds 99%. Similarly, the proposed method provided satisfactory and competitive results and could be considered for QRS complex detection in the ECG signal.

## Discussion

In summary, a few approaches based on grammatical formalism for ECG signal processing and controls were used. The proposed method confirmed that regular grammar domains could be extended to be applied for negative and positive peaks recognition. The QRS complex is assimilated to a pair of adjacent peaks which satisfy certain criteria of standard deviation and duration. Various parameters were determined, such as the number of QRS complex, the QRS durations, the RR distances, and the standard deviations σRR as well σQRS.

Compared with usual methods, the proposed approach affirmed that the use of grammar can represent the QRS structures efficiently. The syntactic approach can describe different types of ECG signals issued from the standard MIT-BIH arrhythmia database. The average sensitivity (Se) rate of the proposed method was 99.74% and the average specificity (Sp) rate was 99.86%. The average False Detection Rate (FDR) rate and the average False Negative Rate (FNR) rate were 0.26 and 0.14% respectively. These results are interesting and can be further improved by enhancing preprocessing.

We used σRR and σQRS of the RR and QRS distances regularity. We defined a threshold where these two variables would be irregular.

In order to study noise sensitivity, the method was applied on different ECG recordings for different SNR values. The variation of the sensitivity and the specificity rates according to SNR was performed. When the SNR values were greater than 40 dB, the method gave high sensitivity values which exceeded 99%. When the SNR values were lower than 24 dB, the sensitivity value decreased to 90%.

## Conclusion

In this paper, the DFA proved useful for QRS complex recognition and ECG signal interpretation. A QRS complex is assimilated to a pair of adjacent peaks that satisfy certain criteria of standard deviation. This method recognizes the QRS complex in an ECG waveform. The QRS complex is described using deterministic automata and regular expressions. For an input signal, all the various indicators such as the complex-QRS durations, the RR distances, the σRR and the σQRS were deduced. The σRR and σQRS parameters were added to quantify the regularity of the RR distances and QRS durations, respectively. This work is aimed at assisting medical diagnosis and providing clinical decision aid for ECG analysis.

Currently, we are working on improving preprocessing and we will propose other grammatical rules to represent distinct pathological cases. We are also working on a hybrid method based on grammar and statistics to ensure a good performance in all cases. The σRR and σQRS variables will be better analyzed on a large scale population in order to provide a fine classification of pathologies.

## Abbreviations

ECG: electrocardiogram
QRS: The sequence of Q, R, and S peaks
DFA: deterministic finite automata
RR: distance separating two consecutive R peaks
TP: True Positive
FP: False Positive
FN: False Negative (FN)
Se: Sensitivity
Sp: Specificity
FDR: False Detection Rate
FNR: False Negative Rate (FNR)
SNR: Signal to Noise Ratio
